# Network analysis of inter-organizational relationships and policy use among active living organizations in Alberta, Canada

**DOI:** 10.1186/s12889-017-4661-5

**Published:** 2017-08-09

**Authors:** Christina C. Loitz, Jodie A. Stearns, Shawn N. Fraser, Kate Storey, John C. Spence

**Affiliations:** 1 Alberta Centre for Active Living, Faculty of Physical Education and Recreation, University of Alberta, Edmonton, Canada; 2 Chronic Disease Prevention – Healthy Living, Population, Public and Indigenous Health, Alberta Health Services, 242, 2nd Floor, WSP Plaza, 10909 Jasper Avenue, Athabasca, AB T5J 4J3 Canada; 3 Faculty of Physical Education and Recreation, University of Alberta, Edmonton, Canada; 4 Faculty of Health Disciplines, Athabasca University, Athabasca, Canada; 5 School of Public Health, University of Alberta, Edmonton, Canada

**Keywords:** Physical activity, Organization, Health promotion, Network analysis, Funding, Partnership, Coordination, Integration, Policy, Active Canada 20/20, Active Alberta

## Abstract

**Background:**

Coordinated partnerships and collaborations can optimize the efficiency and effectiveness of service and program delivery in organizational networks. However, the extent to which organizations are working together to promote physical activity, and use physical activity policies in Canada, is unknown. This project sought to provide a snapshot of the funding, coordination and partnership relationships among provincial active living organizations (ALOs) in Alberta, Canada. Additionally, the awareness, and use of the provincial policy and national strategy by the organizations was examined.

**Methods:**

Provincial ALOs (*N* = 27) answered questions regarding their funding, coordination and partnership connections with other ALOs in the network. Social network analysis was employed to examine network structure and position of each ALO. Discriminant function analysis determined the extent to which degree centrality was associated with the use of the Active Alberta (AA) policy and Active Canada 20/20 (AC 20/20) strategy.

**Results:**

The funding network had a low density level (density = .20) and was centralized around Alberta Tourism Parks and Recreation (ATPR; degree centralization = 48.77%, betweenness centralization = 32.43%). The coordination network had a moderate density level (density = .31), and was low-to-moderately centralized around a few organizations (degree centralization = 45.37%, betweenness centrality = 19.92%). The partnership network had a low density level (density = .15), and was moderate-to-highly centralized around ATPR. Most organizations were aware of AA (89%) and AC 20/20 (78%), however more were using AA (67%) compared to AC 20/20 (33%). Central ALOs in the funding network were more likely to use AA and AC 20/20. Central ALOs in the coordination network were more likely to use AC 20/20, but not AA.

**Conclusions:**

Increasing formal and informal relationships between organizations and integrating disconnected or peripheral organizations could increase the capacity of the network to promote active living across Alberta. Uptake of the AA policy within the network is high and appears to be facilitated by the most central ALO. Promoting policy use through a central organization appeared to be an effective strategy for disseminating the province-level physical activity policy and could be considered as a policy-uptake strategy by other regions.

## Background

The promotion of active living is an important aspect of population health that supports the integration of physical activity into all aspects of life [1, 2]. Unfortunately, levels of physical activity have decreased in most developed countries over the past 50 years [3] with only 15% of adult Canadians meeting the Canadian Physical Activity Guidelines [4]. The causes of physical inactivity are multi-level and complex [5]. To promote physical activity at a population level, the *Toronto Charter for Physical Activity* calls for the development and implementation of a national plan and policies that support physical activity participation [6]. Such physical activity policies have been developed in Canada. For instance, Active Canada 20/20 (AC 20/20) is a change strategy and agenda to increase physical activity and decrease sedentary behaviour levels of Canadians through coordinated actions [1, 2]. Provincial policies and plans such as Active Alberta (AA) also are in place. However, evidence of physical activity policy implementation appears to be poor on a global level [7]. Further, many of these policies often require non-traditional cross-government collaboration and partnerships with organizations from sectors other than health (e.g., transportation, recreation, education) [8].

Inter-organizational partnerships and collaborations are important for optimizing the efficiency and effectiveness of service and program delivery, policy implementation, and the capacity to solve large public health problems [6, 9, 10]. Accordingly, the Toronto Charter and AC 20/20 recognize the importance of partnership and collaboration among various sectors. Unfortunately, limited information is available on the extent to which organizations work together to promote physical activity in Canada [11–13], and even less is known about how policy initiatives are disseminated and taken up [12, 14].

Network mapping and analysis are useful tools for exploring and visualizing relationships between organizations to advance the understanding of complex systems [15]. For instance, collaboration among organizations in a network can optimize availability of resources and expertise, efficiency and effectiveness of services, and capacity to solve difficult problems [9]. Organizational social network analysis involves a set of theories and tools for understanding relationships between organizations, such as collaborations and partnerships [16]. These techniques create a snapshot of existing connections in a network to identify strengths, gaps and opportunities for improvement. Thus, an assessment of the overall structure of the network, along with the position of each organization in the network (e.g., being central or on the periphery) can be used to improve the efficiency of the network in promoting active living [17]. Further, an assessment of physical activity policy use can provide information on the success of policy uptake in the network. Central players in the network (i.e., often called “opinion leaders”) tend to be early adopters of innovations [16]. They are also in a position of power and visibility and thus their uptake of new ideas and behaviours tends to accelerate diffusion through the network [16]. Therefore, if central organizations adopt physical activity policies, we would expect efficient diffusion to other organizations in the network. An examination of the centrality of organizations in relation to their policy use could provide insight on how the physical activity policies were disseminated and how to increase their uptake in the network. It could also help inform the dissemination of physical activity policy in other regions around the world.

Therefore, this project sought to provide a snapshot of the current network of provincial organizations that promote physical activity (i.e., active living organizations [ALOs]) across one Canadian province (i.e., Alberta) and to map out and evaluate the funding, coordination and partnership relationships between organizations. The specific aims of this project were to: 1) examine the overall structure of the funding, coordination and partnership networks of ALOs; 2) examine the most central and isolated ALOs in the funding, coordination and partnership networks; 3) assess the awareness, use and intended use in the future of national (AC 20/20 strategy [1, 2]) and provincial (Active Alberta [18]) physical activity strategies among ALOs; and, 4) examine whether organization centrality (number of connections an organization has with other organizations) discriminates between those who use the AA and the AC 20/20.

## Methods

### Procedure

The study was completed in two stages. First, members of the ALO network and the operational definition of active living were identified through a consensus process. Second, inter-organizational relationships and policy use of the ALO in the network were determined through a network analysis. A university research ethics board approved this study and all participants provided informed consent.

### Sample selection

Five active living researchers and eight ALO leaders (*n* = 13) were recruited to establish the definition of active living and to identify relevant provincial organizations. Key informants completed two concise electronic surveys over a 4-week period. In the first survey, participants rated their agreement with and proposed changes to the following active living definition: “Active living is a way of life in which physical activity is valued and integrated into daily routines for all people” [19, 20]. Next, respondents rated the extent to which 46 identified organizations promote active living provincially. Furthermore, respondent-driven sampling was employed to identify additional organizations [21]. Organizations with an agreement rating of 50% or greater were considered ALOs. Other organizations were evaluated on a case-by-case basis by the research team. The active living definition was accepted by all key informants with 24 of the potential 46 organizations being considered as ALOs. A follow-up assessment of unfamiliar organizations was conducted by the research team; only one of these organizations was kept. In addition, two new ALOs were identified that matched the network criteria. This resulted in a network of 27 ALOs.

### Measures

#### Demographic variables

An online survey was completed between May and July of 2014 by all ALOs (*n* = 27). The survey included individual-level (i.e., sex, age, education, role of respondent, primary area of work) and organizational-level (i.e., primary area and purpose of work, type of organization, role, head office location, and number of employees) demographic questions.

#### Network variables

Network analysis questions assessed the relationships between ALO dyads [22, 23]. In network analysis (also known as a social relations model [24]), each organization is presented with a list of every other organization (*n* = 26) in the network and are asked to specify their relationship (e.g., degree to which they work together) with each organization. This means there are two scores expressing the relationship between each pair of organizations represented in a matrix.

#### Funding flow

Each organization reported the existence and direction of funding flow (response options included: send, receive, both send and receive, neither send or receive, don’t know, not applicable) with every other organization (*n* = 26). These relationships are depicted in the funding network map. For the network analysis, the funding responses were dichotomized as having a funding relationship (send and/or receive) or no funding relationship.

#### Level of integration

Each organization reported the degree of integration (response options: fully integrated, partnership, collaboration, coordination, cooperation, communication, and not integrated; see Table 1 for definitions [17, 25]) with every other organization (*n* = 26). For this study, we focused on partnerships (i.e., work together as a formal team with specified responsibilities to achieve common goals) and coordination (i.e., work side by side as separate organizations to achieve common program goals) relationships. The coordination network reflected coordinated relationships (or a higher level of integration) and non-coordinated relationships between ALOs. The partnership network reflected partnership relationships (or a higher level of integration) and non-partnership relationships between ALOs.

**Table 1:** Defining the Degree of Inter-organizational Integration

Level of Integration	Definition	Coordination Network	Partnership Network
Fully Integrated	We mutually plan, share staff or funding resources and evaluate activities to accomplish our common goals.	Yes	Yes
Partnership	We work together as a formal team with specified responsibilities to achieve common program goals (note: responsibility for each organization is usually outlined in a Memorandum of Understanding or other agreement).	Yes	Yes
Collaboration	We work side-by-side and actively pursue opportunities to work together as an informal team (i.e., do not establish a formal agreement; work together “in the spirit of collaboration”).	Yes	No
Coordination	We work side-by-side as separate organizations to achieve common program goals (i.e., efforts are organized to prevent overlap, but tasks are performed as separate organizations).	Yes	No
Cooperation	We share information and work together when any opportunity arises.	No	No
Communication	We share information only when it is advantageous to either or both programs.	No	No
Not Integrated	We do not work together at all and have separate program goals.	No	No

For the network analysis, the dichotomous variable partnership refers to a partnerships or a fully integrated inter-organizational relationship between organizations. The dichotomous variable coordination refers to a relationship at the coordination level or a greater degree of integration between organizations

#### Policy use

A series of questions regarding awareness, use and intentions to use the AA policy and AC 20/20 strategy were posed (response options: yes, no, not sure).

### Analysis

Pairs of scores were assessed for percent agreement and reliability for funding and integration (coordination and partnership) variables using R 3.1.0 functions [26] for dichotomous and ordinal data [27, 28]. Second, reliabilities were calculated for partner and actor effects using R functions [29] accounting for the round robin design in a social relations model [24]. Partner effects reliability represents the consistency of an organization being rated by other organizations whereas actor effect reliability represents the consistency of each organization in making ratings across the different organizations [30].

Network analysis was completed in UCINET version 6.516. First, the funding, coordination and partnership matrices were symmetrized. Specifically, discrepancies were consolidated by using the highest value response, assuming that the organization reporting the closer relationship had more information about the organization’s funding and level integration with other organizations.

To examine the overall structure of the funding, coordination and partnership ALO networks (aim 1), density, degree and betweenness centralization scores were calculated.

1. *Network density* is the degree of interconnectedness in the network, which is calculated as the number of connections compared to the total possible number of connections [31]. Values range from 0 to 1, with 0 indicating no connections and 1 indicating all organizations are connected.
2. *Degree centralization* assesses the degree to which the network is influenced by one or a few organizations according to the number of direct connections with other organizations. A higher score indicates the network is influenced by only a few organizations, and a lower score indicates that organizations within the network have a similar number of ties.
3. *Betweenness centralization* expresses the degree to which a few organizations have control over the relationships of other organizations in the network. A higher betweenness centralization score indicates greater centralization and a smaller number of gatekeepers that dominate the network.

To identify the most central and isolated ALOs in the funding, coordination and partnership networks (aim 2), degree and betweenness centrality scores were calculated for each ALO and disconnected ALOs (i.e., isolates) were identified.

1. *Degree centrality* score refers to the number of connections each organization has with others.
2. *Betweenness centrality* refers to the number of times an ALO connects other organizations that would not otherwise be connected [32]. Organizations with high betweenness centrality scores act as gatekeepers in the network [32].

We used Valente and colleagues’ [10] “Goldilocks Principle” as a guide for interpreting density, degree centralization and betweenness centralization scores. Specifically, scores below 0.30 were deemed as low (consider increasing), levels between 0.30 and 0.50 as moderate (optimal level), and levels above 0.50 as too high. However we also took the context (e.g., network size) and type of network into consideration (e.g., the partnership network will inherently have lower scores than the coordination network). To help identify connections, strengths, gaps and opportunities in the network, Netdraw version 2.139 was used to map and visually depict the findings.

The remainder of the analyses were completed using IBM SPSS Statistics 23. To assess the awareness, use and intended use of the AA policy and the AC 20/20 strategy (aim 3), frequency scores were calculated. To examine whether organizational centrality in the funding and coordination networks discriminates between those who use the AA policy and the AC 20/20 strategy (aim 4), discriminant function analysis was employed to predict group membership (use or non-use of policy) according to the prominence of network connections (degree centrality score). Separate analyses were run for organizational use or non-use of the AA and AC 20/20 and for the funding and coordination networks, resulting in a total of four discriminant analyses.

## Results

A summary of organizational characteristics is reported in Table 2. The primary area of work for most ALOs was health (22%), recreation (22%) and education (33%), and the primary purpose of work for most ALOs was program delivery (37%), services (15%), funding (15%) and education (15%). Most were not-for-profit organizations (56%). The reliability results between the organizational dyads are reported in Table 3. A high degree of reliability was observed for the funding and partnership dyads, with the coordination dyads showing a good percent agreement and moderate level of inter-rater reliability. However, the partner and actor effects reliability for coordination was quite good. Overall reliability among raters was good.

**Table 2:** Summary of Organizations’ Characteristics (*N* = 27)

Variable	*M* (range)	Response category	*n*	%
Primary area of work		Transportation Fitness Child services or programming Human services Community Health Recreation Education	1 1 1 1 2 6 6 9	4 4 4 4 7 22 22 33
Primary purpose of work		Certification Knowledge translation Other or missing Education Funding Providing services Program delivery	1 2 2 4 4 4 10	4 7 7 15 15 15 37
Type of organization		Private sector Government Non-government Not-for-profit	1 4 7 14	4 15 26 56
Location of main office		No main office Calgary Edmonton	1 4 22	4 15 82
Which other cities is there a branch		Medicine Hat Red Deer Fort McMurray Grand Prairie Lethbridge Calgary Edmonton	7 8 8 10 11 ﻿1721	26 30 30 37 41 63 78
Number of employees	173.22 (0 to 2300)^a^			
Number of employees in active living	80.48 (0 to 2000)^a^			

^a^Organizations led and run by volunteers and contractors reported 0 employees

**Table 3:** Inter-rater Reliability for Network Questions

	Percent Agreement (%)	Gwet’s AC1/AC2	95% CI for AC1/AC2	Actor Effects	Partner Effects
Funding	92.3	.90	.85 to .94	.78	.74
Coordination	70.2	.54	.38 to .70	.84	.84
Partnerships	84.1	.80	.72 to .89	.77	.75

Results are weighted for percent agreement and Gwet’s AC1 is reported for Coordination and Partnerships, whereas Gwet’s AC2 is reported for Funding

### Funding flow

**Fig. 1:**
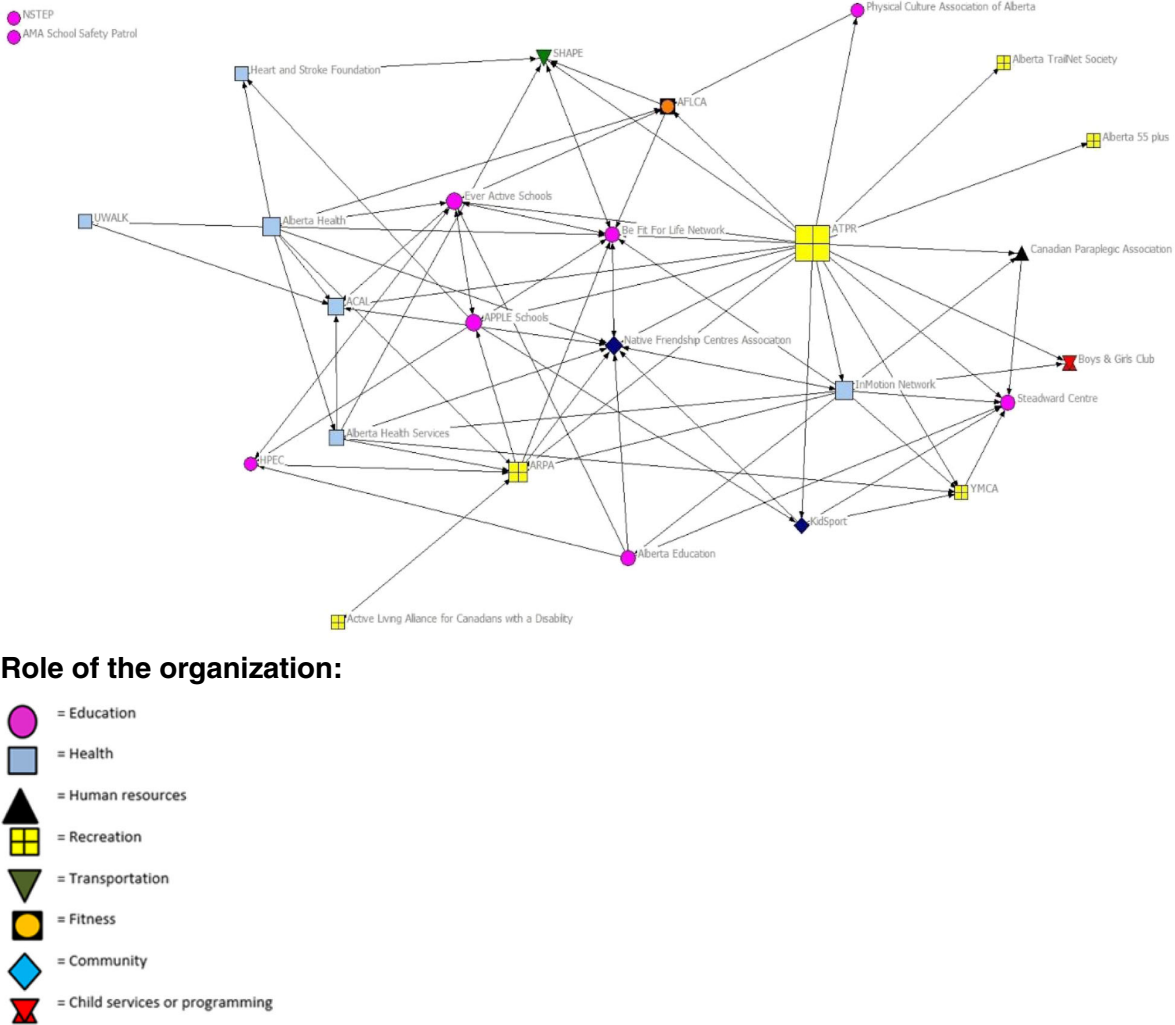
Network map of funding flow between active living organizations in Alberta. Note: The size of the node is relative to the degree of betweenness centrality score

The funding network results are presented in Table 4 and depicted in Fig. 1. The network density score of .20 indicates a low interconnectedness of funding relationships. Degree centralization was 48.77%, indicating the funding network was moderately centralized around a few organizations. The betweenness centralization score of 32.43% identified that there was a moderate level of gatekeepers across the network. ALOs had between 0 and 17 funding connections (i.e., degree centrality), with an average of five funding connections. Alberta, Tourism, Parks and Recreation (ATPR) had the largest number of funding ties (17 ties) in the network, followed by the InMotion Network (10 ties). ATPR had the highest betweeness centrality score (112.06), a score three times greater than any other ALO, which means they are a key gatekeeper in the funding flow network. Two isolates were identified. A visual inspection of the network and these statistics show that ATPR’s numerous direct ties with organizations and their bridging of ties between organizations made them the most powerful organization within the funding network.

**Table 4:** Network Density and Centralization Scores

Network	Network density	Degree centralization (%)	Organizations with highest degree centrality scores		Betweenness centralization (%)	Organizations with highest betweenness centrality scores	
			ALO	Unnormalized score (normalized score)		ALO	Unnormalized score (normalized score)
Funding	.20	48.77	ATPR IMN ARPA, AH, BFFL & NFC	17 (0.65) 10 (0.39) 9 (0.35)	32.43	ATPR ARPA AH	112.06 (34.48) 33.32 (10.25) 20.93 (6.44)
Coordination	.31	45.38	ATPR AHS BFFL	19 (0.73) 17 (0.65) 16 (0.62)	19.92	ATPR AHS ARPA	71.20 (21.91) 32.34 (9.95) 26.97 (8.30)
Partnership	.15	54.00	ATPR EAS AHS	17 (0.65) 10 (0.39) 9 (0.35)	40.88	ATPR EAS SHAPE	139.75 (43.00) 34.68 (10.67) 30.75 (9.46)

Betweenness centralization is presented as the Network Centralization Index. *AH* Alberta Health, *AHS* Alberta Health Services, *ARPA* Alberta Recreation Parks Association, *BFFL* Be Fit for Life, *EAS* Ever Active Schools, *NFC* Native Friendship Centres, *IMN* InMotion Network, *SHAPE* Safe Healthy Active People Everywhere

### Coordination – Integration

**Fig. 2:**
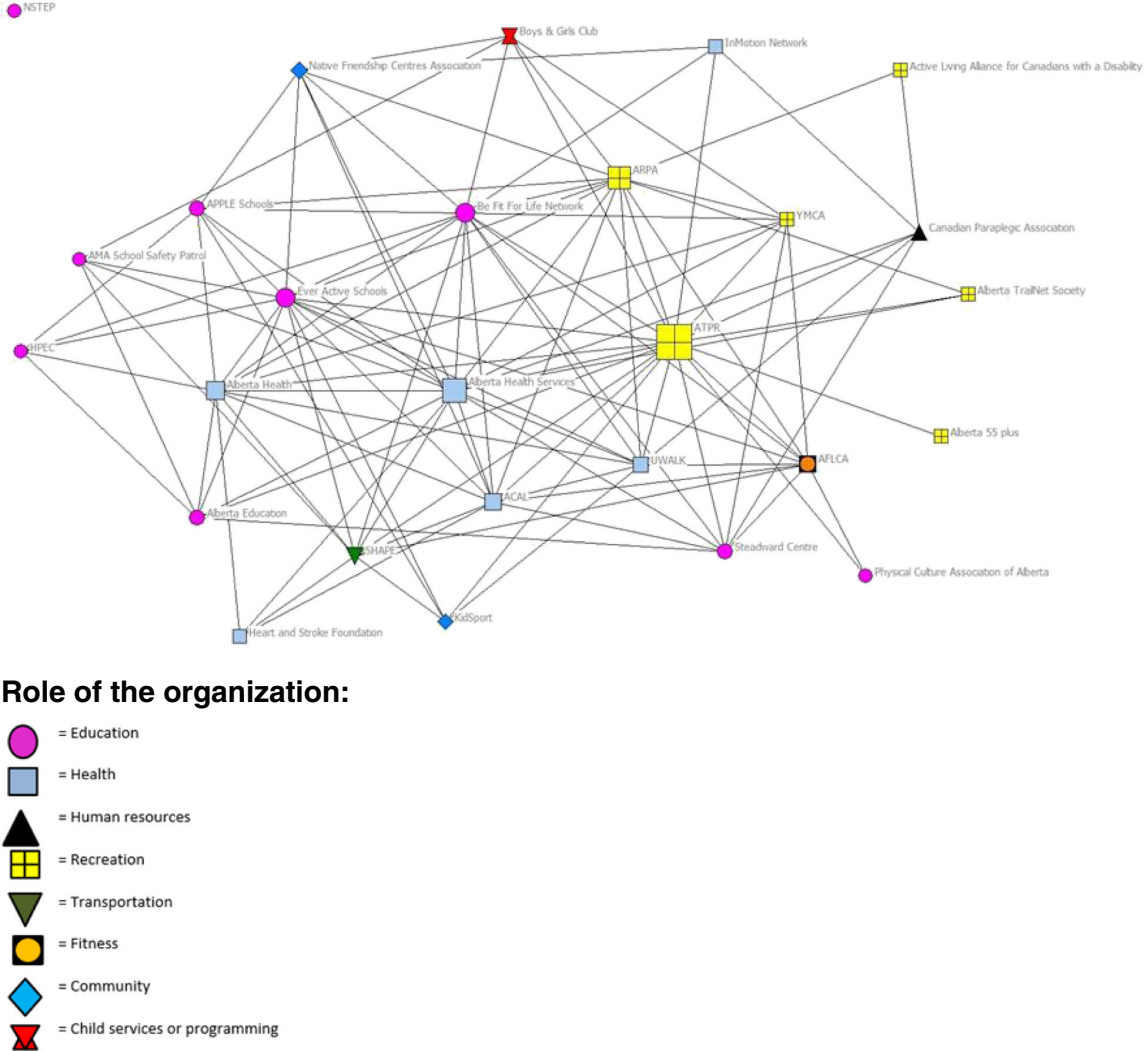
Network map of coordination between active living organizations in Alberta. Note: The size of the node is relative to the degree of betweenness centrality score

Inter-organizational coordination involved two organizations working side by side to achieve common goals [17]. The results from the coordination network analysis are presented in Table 4 and Fig. 2. The coordination network density score was the highest (0.31) of the three networks, this score is considered a moderate level of density. Degree centralization was 45.38% indicating the coordination network was moderately centralized around a small group of organizations. The betweenness centralization score of 19.92% indicates a low degree of centralization, and a network that is not centered on a small number of gatekeepers. On average the organizations coordinated with eight other organizations, and the number of coordinated relationships ranged from 0 to 19 across the network. ATPR had the largest number of coordination ties (19 ties) in the network, followed by Alberta Health Services (17 ties) and Be Fit for Life (BFFL, 16 ties). There was one isolate in the coordination network.

### Partnership – Integration

**Fig. 3:**
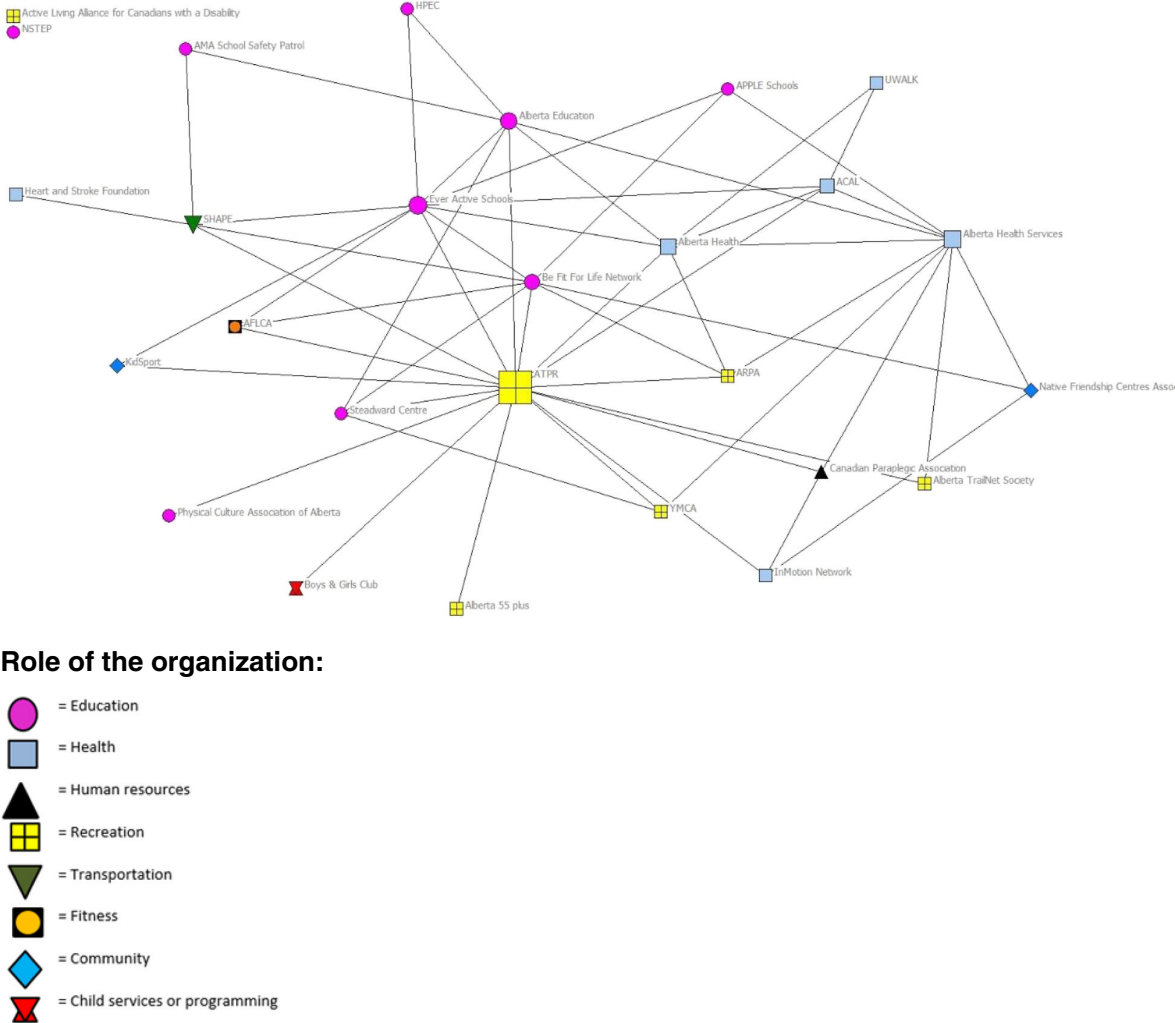
Network map of partnership between active living organizations in Alberta. Note: The size of the node is relative to the degree of betweenness centrality score

Partnerships involved two or more organizations formally working together with specified responsibilities to achieve a common goal. Results from the network analysis are presented in Table 4 and Fig. 3. The partnership network was loosely connected with a low network density score (.15). Degree centralization was 54.00%, which indicates the network was highly dominated by a few organizations. According to the betweenness centralization score of 40.88%, this network included a moderate level of gatekeeper organizations. On average, organizations had four partnerships; however the number of partnership connections ranged from 0 to 17. ATPR was the key gatekeeper in the partnership network (17 ties), followed by Ever Active Schools (EAS, 10 ties). There was one isolate in the partnership network.

### Policy awareness and use

The provincial policy and national strategy (AA and AC 20/20) were used by many of the ALOs. The online survey identified that 88.46% of the respondents had heard of the AA policy; 66.67% were using the policy; and 84.62% planned to use the policy in the future. The respondents were less familiar and experienced with the AC 20/20 strategy. Though 77.78% of the organizations had heard of AC 20/20, only 33.33% were using the strategy, and 50.00% planned to use the strategy in the future.

### Predicting policy use from degree centrality

Sample size was acceptable and centrality scores met the assumptions of discriminant analysis. The degree of centrality of the funding network predicted the use of the AA policy, F(1,27) = 5.94, *p* = .03, Wilks’ Lambda = .80; squared canonical correlation = .21. ALOs with more funding connections were more likely to use the AA policy than those with less funding connections. The mean number of ALO funding connections was 6.39 (SD = 3.97) for ALOs that used AA and 3.00 (SD = 2.45) for ALOs that did not use AA. Degree centrality in the coordination network was not a significant predictor of AA policy use F(1,27) = 2.40, *p* = .14, Wilks’ Lambda = .92; canonical correlation squared = .09. Degree centrality within the funding network significantly predicted use of AC 20/20, F(1,27) = 5.03, *p* = .03, Wilks’ Lambda = .83; canonical correlation squared = .17. The mean number of ALO funding connections was = 7.44 (SD = 4.67) for ALOs that used AC 20/20 and 4.17 (SD = 2.94) for ALOs that did not use AC 20/20. Additionally, level of centrality in the coordination network significantly predicted use of AC 20/20 F(1,27) = 9.32, *p* = .005, Wilks’ Lambda = .73; canonical correlation squared = .27. The mean number of ALO coordination connections was 11.78 (SD = 5.47) for ALOs that used the AC 20/20 strategy and 6.22 (SD = 3.89) for ALOs that did not use the AC 20/20 strategy.

## Discussion

Social network methods were used to explore the structure of inter-organizational funding, coordination and partnership relationships among a network of stakeholders with an invested interest in active living. Additionally, the use of the AA policy and AC 20/20 strategy was assessed according to the ALO’s position within the funding and coordination network. The results suggest that integrating organizations with limited connections through formal and informal relationships will benefit these ALOs along with the capacity of the entire network to promote active living. Uptake of the AA policy within the network was high and appears to be facilitated by the most central ALO. There was a lower uptake of AC 20/20, however the most central ALOs were using the policy, thus it may require more time to disseminate further.

### Inter-organizational network structure

Density and centralization were used to assess the inter-organizational structure of the Alberta ALO funding, coordination and partnership networks. Density provided information on the level of inter-organizational connectedness in the network. As anticipated, more organizations worked independently towards similar goals while being aware of others work (coordination), than organizations working together as a formal team (partnership). Specifically, on average Alberta ALOs had funding relationships with 20% (low density) and worked in coordination with 30% (moderate density) and in partnership with 15% (low density) of the other ALOs in the network. There are both advantages and drawbacks to working in a partnership network [9]. Some benefits of such a network include: regular ongoing communication between organizations, more integrated plans and actions towards a common vision and greater access to pooled resources (e.g., funding, human resources). Potential drawbacks to a partnership include: the relatively large amount of time required to maintain relationships, make decisions and formalize agreements, plans and processes. Additionally, groupthink may constrain the development of innovative or unique projects. Furthermore, the vision of the network of partnering organizations may not fit the vision, mission or role of each organization. These variations among organizations may be a barrier to devoting resources towards the partnership. For example, a service delivery organization may prefer to work in coordination rather than partnership with other active living organizations in order for them to spend more efforts on achieving their own organizational objectives. Therefore, though increasing the partnership connections in the network could help increase the capacity of the network to promote active living, some disadvantages may exist for an overly connected network. Obtaining the optimal amount and degree of integration of organizations within the network is an ongoing challenge for organizations involved in these networks. This is further complicated given the dynamic nature of these network connections which and may change in terms of amount and degree over time.

The Alberta ALO coordination network density was similar to eight American state tobacco control programs where densities ranged from .30 to .59 within networks of 11 to 15 organizations [33]. The density of the Alberta funding network was greater than the Hawaiian active living funding network (Hawaiian funding network = .07) of a similar size (Alberta network = 27, Hawaiian network = 26), which included both local-level and state-level organizations [17]. Though density scores provided important information on the interconnectedness of the network as a whole; they do not describe the distribution of the relationships throughout the network [31].

The magnitude to which the network was dominated by a small group of organizations was assessed by degree and betweenness centralization [34]. Networks with a high-degree of centralization are connected by a small group of organizations that are well positioned to develop strong inter-organizational relationships built on trust, and thus are able to quickly disseminate information to the network [35]. Whereas networks with a low degree of centralization have a similar amount of ties across the network, and are better at generating new information and building on diversity [35]. Therefore a moderate level of centralization is ideal (i.e., between .30 and .50) [10], however it is important to take the type of network tie and context into consideration. Degree centralization of the ALO networks were in the moderate to high range (coordination = 45.38, partnership = 54.00, funding = 48.77). The higher degree centralization score for the partnership network is reasonable considering the high level of integration required for partnerships (i.e., working together as a formal team). The betweenness centralization (i.e., the degree to which a few organizations had control over the relationships between other organizations in the network) scores in our study were in the low to moderate range (coordination = 19.92, partnership = 40.88, funding = 32.43). The lower betweenness centralization score for the coordination network is reasonable. Thus overall the degree and betweenness centralization scores in the ALOs are good. In comparison to an obesity prevention network (cooperation = 44.68; collaboration = 23.07) [35], the centralization of the ALO network integration were greater.

We identified ATPR as the betweenness centralization gatekeeper in all networks, connecting several organizations that would not otherwise be connected. When the number of connections were considered (i.e., degree centrality), ATPR had a substantially larger number of funding and partnership connections compared to the other organizations, however several organizations had a large number of coordination connections. Thus, ATPR is an ideal organization for knowledge dissemination.

Coordinated inter-organizational relationships support knowledge exchange informing organizational planning and practices. For example, inter-organizational knowledge sharing within the network can: facilitate the introduction of new organization or organizational leaders to others in the network, assist in the navigation of organizational roles by considering work to fill knowledge or service gaps and avoid duplication, and support other organizations initiatives [9]. A network supporting coordinated and collaborative inter-organizational relationships helps organizations find a niche where they can specialize and contribute to the body of network activities, rather than attempt to be the expert across populations, locations and fields. Thus, taking steps to purposefully and strategically increase the quantity of coordination and partnership relationships within the ALO network may improve the efficiency and effectiveness of current health promotion efforts to promote physical activity, reaching a broader target audience, and increasing access to resources and individuals with specialized skill sets [36].

### Policy use in the ALO network

Physical activity policies and strategies have the potential to instigate action to improve provincial physical activity levels and subsequent population-level health outcomes. Policy assessments are important activities to undertake to gain information on the awareness, acceptance and implementation of policy in practice. The findings from this study identified that the majority of the ALOs were aware of both the national strategy (AC 20/20) and the provincial policy (AA). The latter was used and intended to be used in the future by ALOs, whereas AC 20/20 was not currently being used by many ALOs. Only half the respondents considered using AC 20/20 in the future. Furthermore, organizations with more coordination connections were more likely to use the national AC 20/20 strategy in their work compared to ALOs with fewer coordination connections. However, no difference existed in use or non-use of the provincial AA policy. In the funding network, those with more connections were more likely to use both the AC 20/20 strategy and the AA policy. The unified strategies or policies supporting coordinated efforts across sectors are critical for success in health promotion [37], and important for all ALOs to be aware of and employ. Further efforts to maintain or improve the use of these policies in the ALO network will be important for advancing the physical activity agenda in Alberta and Canada [1, 2].

Networks are often controlled by the most central organizations for the flow of information and resources, which is one source of organizational power [9]. For effective diffusion, network function and policy engagement, the development and dissemination of policy should be led by a central organization. ATPR was consistently the most central organization in the network, which made them a key organization for the dissemination of information surrounding provincial policy and national strategies. The fact that the AA policy was developed by ATPR likely increased communication and collaborative efforts in the provincial ALO network. Additionally, ATPR was one of the provincial representatives engaged in the development and dissemination of AC 20/20 which was designed to support and guide stakeholders at all levels of government [1]. Furthermore, applications for active living funding from ATPR are required to articulate how the proposed projects link to the two policies. These activities contributed to ATPR’s support for, and leadership in increasing the awareness and use of physical activity policy by ALOs in Alberta. Considering the high rates of awareness and use of the AA policy in the network, ATPR’s dissemination of this policy appears to be effective, and shows how dissemination of policy from those central in the network is a valuable strategy. Because AA incorporates many of the principles of AC 20/20, it may not be imperative that provincial organizations use AC 20/20. Other regions in Canada and around the world may want to consider using central leaders in provincial active living organizational networks for facilitating policy uptake in their networks.

### Strengths and limitations

A major strength of the study was the adoption of social network analysis to understand the provincial ALO network, which allowed us to examine the overall structure of inter-organizational relationships. Consultation with key informants on the definition of active living ensured a consensus on this definition and supports the validity of the social network questions. Including the key informants in the establishment of the organizations in the network also ensured all provincially-mandated ALOs in Alberta were included in our survey.

Despite these strengths a few potential limitations should be mentioned. In an attempt to mitigate response biases, a research assistant from outside the network of ALOs was hired to conduct the data collection. Though the research assistant was not a fulltime employee of any of the organizations within the ALO network, she was paid by and followed the protocol designed by one of the organizations within the network. This may contribute to some potential response biases by the ALOs. Further, the reported network analysis represents a snapshot of inter-organizational relationships within the ALO network at a specific time. Thus, we cannot be certain that ATPR was entirely responsible for the uptake of the AA policy and AC 20/20 strategy in the network, and it is likely that use of these policies encouraged the organizations to form relationships. Further, changes are constantly occurring within (staff, funding, focus, vision, leadership and priorities) and between organizations (development and dissolving of relationships), and in the health promotion landscape. Indeed, since this data was collected, ALOs in this network have undergone major changes including loss of funding, shifts in organizational structures, staff and leadership turnover and substantial modifications to organizational vision. Future research will benefit from continuing to monitor the structure and needs of the ALO network over time.

Though network analysis is a useful method of examining the structure of a network, additional information on the effectiveness or state of the network should be gathered to provide a more comprehensive understanding of the network [9]. A mixed-methods approach, including interviews or focus group sessions, would provide a more thorough explanation of the factors that support and impede effective networks. Future research could explore the quality and length of inter-organizational relationships to increase knowledge and plan for success. Additionally, impressions from and needs of the target audiences of the ALO network (e.g., general public, practitioners) could elicit a unique perspective of the current state and future directions for the network [38]. Connections between organizations and the degree of inter-organizational engagement in social media may be examined to learn more about network structure and function. Finally, since local organizations often offer physical activity programs or services, it would be beneficial to explore the role of local organizations within the provincial network in Alberta, especially for the purpose of bi-directional knowledge sharing between ALOs of promising practices from theoretical, literature-based and experiential perspectives.

Moreover, the extent to which these results generalize to other provinces in Canada, or other countries, is not clear since the current assessment was a measure of a specific network at a particular moment in time. However, the findings are consistent with social network theory and empirical evidence showing the benefits of disseminating policy using central organizations. Further, this work does provide a reference point for other communities and a baseline to build and strengthen the ALO network overtime and compare to future assessments of this network. Additionally, findings from the ALO network analysis in Alberta can be used to inform like-networks on the use of policy and structures to build their capacity to address similar health issues.

## Conclusions

Exploring the relationships between organizations that promote physical activity can provide insight into the inter-organizational support for health promotion practices. Network analysis is one method of gathering information to examine the structural relationships among ALOs as well as the use of policy across a network. Network analysis found that ALOs with more funding connections were more likely to use the provincial active living policy (AA) than those with less funding connections; although no relationship existed between the number of inter-organizational coordination connections and policy use. Understanding the barriers and facilitators of inter-organizational relationships and policy use is key in the development, implementation, evaluation and maintenance phases of health promotion interventions focused on complex social and population health issues, such as physical inactivity [39]. Findings from this analysis can be used to improve the effectiveness of the active living promotion system.

## Abbreviations

AA: Active Alberta
AC 20/20: Active Canada 20/20
AH: Alberta Health
AHS: Alberta Health Services
ALO: Active Living Organization
ATPR: Alberta Tourism Parks and Recreation
BFFL: Be Fit For Life
EAS: Ever Active Schools
IMN: InMotion Network
NFC: Native Friendship Centres
SHAPE: Safe Healthy Active People Everywhere
